# Synthesis of Some Novel 2-Amino-5-arylazothiazole Disperse Dyes for Dyeing Polyester Fabrics and Their Antimicrobial Activity

**DOI:** 10.3390/molecules21010122

**Published:** 2016-01-21

**Authors:** Hatem E. Gaffer, Moustafa M. G. Fouda, Mohamed E. Khalifa

**Affiliations:** 1 Textile Research Division, National Research Center, Dokki, Giza, P. O. Box.12611, Egypt; hatem197@yahoo.com (H.E.G.); m_gaballa@yahoo.com (M.M.G.F.); 2 Chemistry Department, College of Science, King Saud University, P. O. Box 2455, Riyadh 11451, Saudi Arabia; 3 Chemistry Department, Faculty of Science, Taif University, P. O. Box 888, Taif 21974, Saudi Arabia; 4 Department of Chemical Engineering, Higher Institute for Engineering and Technology, New Damietta, P. O. Box 34518, Egypt

**Keywords:** azo dyes, 2-aminothiazole, sulfaguanidine, polyester, dyeing, antimicrobial activity

## Abstract

The present work describes the synthesis of a series of four novel biologically active 2-amino-5-arylazothiazole disperse dyes containing the sulfa drug nucleus. The structures of the synthesized thiazole derivatives are confirmed using UV-spectrophotometry, infrared and nuclear magnetic resonance techniques and elemental analysis. The synthesized dyes are applied to polyester fabrics as disperse dyes and their fastness properties to washing, perspiration, rubbing, sublimation, and light are evaluated. The synthesized compounds exhibit promising biological efficiency against selected Gram-positive and Gram-negative pathogenic bacteria as well as fungi.

## 1. Introduction

Azo dyes are the most essential group of disperse dyes [1,2] and universally constitute more than 50% of disperse dyes due to their strong tinctorial strength, ease of synthesis and low cost of manufacture. Azo dyes have vivid colors in the yellow to blue-green region adjustable by varying between azo components with electron withdrawing groups and coupling components with electron donating groups. 2-Aminothiazoles have considerable interest from a therapeutic point of view because of their utility as intermediates in the synthesis of antibiotics such as the well-known sulfa drugs [3]. They have been reported in the literature as antimicrobial [4], antifungal [5], anti-inflammatory activity [6], anesthetic [7], anti-hypertesive [8] and antiviral drugs [9]. 2-Aminothiazole derivatives have been also used as intermediates in the synthesis of various types of dyes [10,11,12,13]. In continuation of our interest in the synthesis of arylazothiazole derivatives [14,15,16], this paper reports the synthesis of some 2-amino-5-arylazothiazoles and their application as disperse dyes on polyester fabrics. In addition, the antibacterial activities of the synthesized dyes against various pathogenic bacteria and fungi were also investigated.

## 2. Results and Discussion

### 2.1. Synthesis

**Scheme 1:**
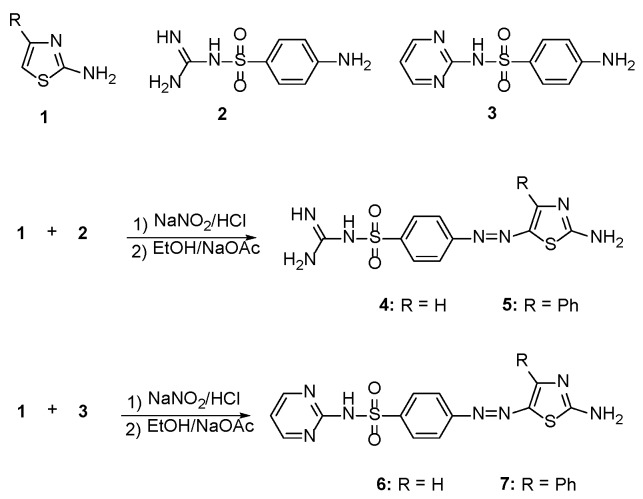
Synthesis of 2-amino-4-arylazo-thiazole disperse dyes **4**–**7**.

Treatment of 2-aminothiazole derivatives **1** with the diazonium chloride derived from sulfaguanidine **2** afforded the corresponding 2-amino-5-arylazothiazole derivatives **4** and **5** as shown in Scheme 1. The structures of **4** and **5** were established on the basis of their elemental analyses and spectral data. For example, the IR spectrum of **4** revealed absorption bands at 3346, 3232, 3173 cm^−1^ due to NH_2_ and NH functions, while the absorption band at 1610 cm^−1^ was attributed to the C=N function. The ^1^H-NMR spectrum displayed a triplet signal at δ 7.05 ppm due to the NH proton, two doublet signals at δ 7.74 and 8.04 ppm corresponding to the aromatic protons, a singlet at δ 8.16 ppm characteristic for the C-4 thiazole-H, two signals at δ 8.51 and 8.67 due to two NH_2_ groups and a singlet signal at δ 11.88 ppm due to NH proton.

Moreover, coupling of 2-aminothiazole derivatives **1** with the diazonium chloride derived from sulfapyrimidine **3** furnished the corresponding 2-amino-5-arylazothiazole derivatives **6** and **7** as shown in Scheme 1. The structures of these substituted thiazole dyes **6** and **7** were elucidated based on their elemental analyses and spectral data. The IR of the dye **6** showed absorption peaks at 3364, 3286, 3142 cm^−1^ due to the presence of NH_2_ and NH functions, in addition to the characteristic C=N group absorption peak at 1612 cm^−1^. The ^1^H-NMR spectrum of dye **6** showed a triplet signal at 6.88 ppm due to C-5 pyrimidine proton, a singlet at 7.13 ppm for the NH group, two doublets at 7.66 ppm and 8.04 ppm for four aromatic protons and a singlet at 8.22 ppm for the C-4 thiazole proton. The presence of a doublet at 8.40 was attributed to the C-4 and C-6 pyrimidine-H protons, while the NH_2_ group appeared shifted to 8.70 ppm.

### 2.2. Dyeing and Fastness Properties

The functionalized 4-arylazo-3-methyl-2-substituted-thiophene disperse dyes **4**–**7** were applied to polyester fabrics by 2% shade on the weight of fabric (o.w.f.), using the high-temperature pressure technique (130 °C), and the visual color shades varied from red to reddish violet. Dyeing of polyester fabrics was assessed in terms of their fastness properties (e.g., fastness to washing, perspiration, rubbing, sublimation, and light) via a standard method [17]. All results, provided in Table 1, reveal that these dyes have good fastness properties to washing, perspiration, rubbing and sublimation.

The light fastness of the synthesized dyes on polyester showed fading which is significantly affected by the presence of the azo group, which decomposes by oxidation, reduction and/or photolysis [18]. The rate of this process depends on the chemical structure, treatment conditions, as well as the nature of the substrate. The use of polyester fabric (*i.e.*, non-proteinic fabric type), explained that the fading process likely occurred by oxidation [19]. The ease of oxidation of azo linkages should be a function of their electron density, thus the presence of electron-donating substituents should increase the fading rate. This proposal was in agreement with the observed results (Table 1) which demonstrate that the presence of the attached phenyl group decreased of light fastness to 4–5 (dyes **5** and **7**).

**Table 1:** Fastness properties of the synthetic dyes on polyester fabrics.

Dye	Washing ^1^	Perspiration ^1^		Rubbing ^1^		Sublimation ^1^		Light ^2^ (60 h)
		Acid	Alkali	Dry	Wet	Staining at 180 °C	Staining at 210 °C	
**4**	4–5	4–5	4–5	3–4	4–5	4	3	5
**5**	4–5	3–4	4–5	3	3	5	4	4–5
**6**	4–5	4–5	4–5	3–4	4–5	5	4	5
**7**	4–5	4	4	4	4	3	3	4–5

^1^ These properties were evaluated using the grey scale (1—poor, 2—fair, 3—moderate, 4—good and 5—excellent). ^2^ This property was evaluated using the blue scale (1, 2—very poor, 3—poor, 4—fair, 5—good, 6, 7—very good, 8—excellent) [17].

### 2.3. Color Assessment

Colors of polyester fabrics is expressed in terms of CIELAB values (Table 2) and the following CIELAB coordinates are assessed: lightness (L*), chroma (C*), hue angle from 0° to 360° (H), (a*) value exemplifies the degree of redness (positive) and greenness (negative) and (b*) signifies the degree of yellowness (positive) and blueness (negative). A reflectance spectrophotometer (Gretag Macbeth CE 7000a) was utilized for the colorimetric assessments of the dyed samples. K/S values provided by the reflectance spectrometer were calculated at λ_max_ and were directly associated with the dye concentration on the dye substrate.

**Table 2:** Optical measurements of the synthetic dyes on polyester fabrics.

Dye	%Exhaustion	Absorption λ_max_/nm	K/S	L*	a*	b*	C*	H
**4**	65	446	9.26	81.09	3.35	20.42	40.58	80.71
**5**	71	444	12.67	73.23	−1.48	23.13	43.88	76.75
**6**	69	463	11.33	74.22	8.17	37.20	48.71	76.21
**7**	66	458	9.37	89.35	−1.78	3.14	43.51	85.84

Generally, the color hues of the thiazole dyes **5** and **7** on polyester fabric shifted to the greenish directions, this was expressed by the negative values of a* (red-green axis). The positive values of b* (yellow-blue axis) designated that the color hues of the thiazole dyes **4**–**7** on polyester fabric are shifted to the yellowish directions as shown in Table 2.

**Figure 1:**
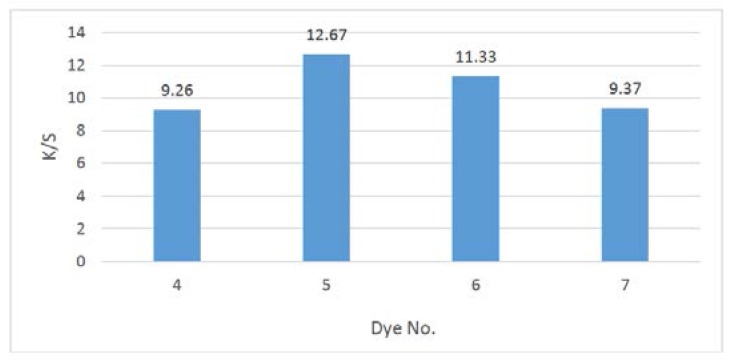
Comparison of K/S values for the synthesized disperse dyes **4**–**7** series.

Application of the synthesized compounds **4**–**7** as disperse dyes, showed the good affinity of such dyes to polyester fabrics, as indicated from the satisfactory color yields and the acceptable K/S values. Figure 1, illustrated the relationship between the dyeing bath concentrations and the strengths of the synthesized disperse dyes **4**–**7**, under high temperature dyeing conditions, where no direct connection between the relative molecular weight of these dyes to the build-up was clear. These results were in line with the previously reported by Müller [20] on the relation between substituents and dye structure.

### 2.4. Antimicrobial Activity

Table 3 shows that all samples exhibited an acceptable antimicrobial activity against the tested pathogenic bacteria at different concentrations 0.01, 0.5 and 1 μg/mL, where all the results are mean of three replicates and significant difference at *p* < 0.05 by student’s test. At high concentrations, regarding *C. albicans*; however, the extract exhibited an acceptable antifungal activity. The inhibition zone of all synthetic products was at range 0.6 to 1.7 mm. The remarkable activity of the synthesized compounds **4**–**7** against Gram-negative bacteria, which were known to be more resistant against antibiotics than Gram-positive bacteria because of their impenetrable cell wall, should be undergo further studies in order to understand well the mechanism of action for each active compound.

### 2.5. Scanning Electron Microscopy

**Figure 2:**
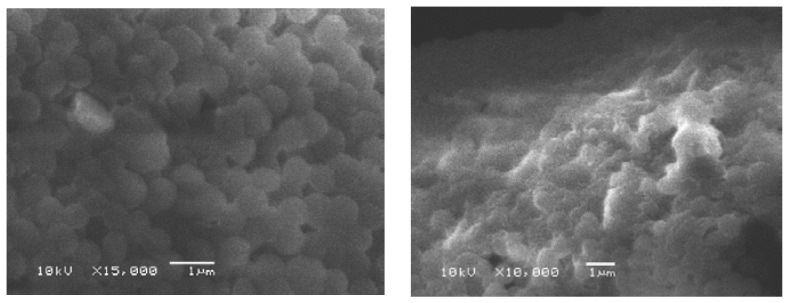
*S. aureus* in ethanol as control (**left**); *S. aureus* treated with synthesized compound **7** (**right**).

**Figure 3:**
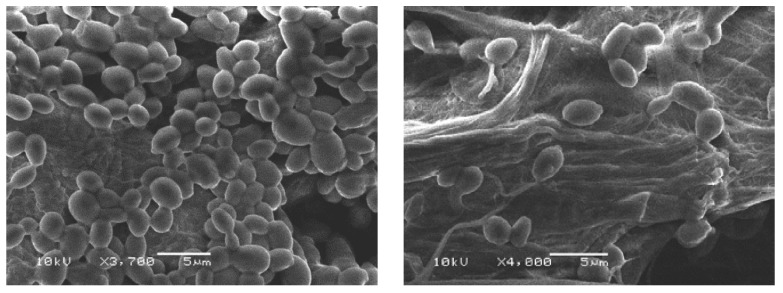
*Candida albicans* in ethanol as control (**left**); *Candida albicans* treated with synthesized compound **7** (**right**).

Scanning electron microscopy was used to observe the morphological alteration of the cells after treatment with the synthesized compounds (e.g., the most active compound **7**). As shown in Figure 2 and Figure 3, the bacterial (*S. aureus*) and fungal (*Candida albicans*) cells treated with the synthesized compound appeared to be shrink and the cell wall degradation was observed.

### 2.6. Minimum Inhibitory Concentration (MIC)

A strong inhibition of the synthesized compounds **4**–**7** against the tested pathogenic bacteria was noticed (5 to 150 μg/mL) as described in Table 4, to be vulnerable to all of them and their minimum inhibition concentrations (MIC) values were ranged from 5–10 μg/mL, with noticeably inhibitory action. In this study, synthesized compounds **4** and **7** showed the highest antibacterial activity against the tested bacteria with the lowest MIC value of 5 μg/mL. According to the obtained results, it is obviously clear that, all synthesized compounds **4**–**7** exhibited broad-spectrum activity against both Gram-positive bacteria, Gram-negative bacteria as well as fungi.

**Table 3:** Antimicrobial activity expressed as inhibition diameter zones (mm) of the synthesized compounds **4**–**7** at different concentrations (μg/mL).

Compd. No.	Gram-Positive Bacteria									Gram-Negative Bacteria						Fungi	
	*S. aureus*			*S. pyogenes*			*S. typhi*			*P. aeruginosa*			*K. pneumoniae*			*C. albicans*	
	0.01	0.5	1.0	0.01	0.5	1	0.01	0.5	1	0.01	0.5	1	0.01	0.5	1.0	0.005	0.05
**4**	0.6	0.7	1	0.4	0.6	0.65	0.6	0.9	1.2	0.3	0.6	0.8	0.2	0.6	0.7	0.7	1.1
**5**	0.6	0.8	1.2	0.1	0.2	0.6	0.6	0.8	1	0.2	0.4	0.6	0.2	0.6	0.7	0.9	1.3
**6**	0.6	1	1.7	0.3	0.6	1.6	0.6	1	1.2	0.7	1	1.4	0.3	0.5	1.2	0.7	1.4
**7**	0.6	1.2	1.3	0.6	1.1	1.9	0.7	1.2	2	0.1	0.3	0.7	0.4	1	2	0.8	1.3
Ciprofloxacin	0.9	1.2	1.4	0.8	1.0	1.3	0.8	0.9	1.5	0.8	1.0	1.4	0.5	0.8	1.2	--	--
Ketoconazole	--	--	--	--	--	--	--	--	--	--	--	--	--	--	--	0.8	1.4

**Table 4:** Minimum Inhibitory Concentration (µg/mL) of the synthesized compounds **4**–**7** against Gram +ve (e.g., *S. aureus* and Gram -ve (e.g., *S. typhi*) bacteria at different concentrations (μg/mL).

Compd.#	Control Conc. (%)	*S. aureus*				Control Conc. (%)	*S. typhi*			
		5	10	60	150		5	10	60	150
**4**	49.32 × 10^3^ (0 %)	37.65 × 10^3^ (23.7 %)	31.44 × 10^3^ (36.3 %)	24.80 × 10^3^ (49.7 %)	19.39 × 10^3^ (60.7 %)	36.09 × 10^3^ (0 %)	34.22 × 10^3^ (5.2 %)	25.36 × 10^3^ (29.7 %)	22.71 × 10^3^ (37.1 %)	16.64 × 10^3^ (53.9 %)
**5**	49.32 × 10^3^ (0 %)	43.47 × 10^3^ (11.9 %)	26.16 × 10^3^ (47.0 %)	25.22 × 10^3^ (48.9 %)	17.32 × 10^3^ (64.9 %)	36.09 × 10^3^ (0 %)	36.56 × 10^3^ (1.3 %)	31.89 × 10^3^ (11.6 %)	22.24 × 10^3^ (38.4 %)	15.56 × 10^3^ (56.9 %)
**6**	49.32 × 10^3^ (0 %)	48.94 × 10^3^ (0.8 %)	48.19 × 10^3^ (1.0 %)	46.31 × 10^3^ (6.1 %)	28.24 × 10^3^ (42.7 %)	36.09 × 10^3^ (0 %)	32.67 × 10^3^ (9.5 %)	20.22 × 10^3^ (44.0 %)	19.44 × 10^3^ (46.1 %)	16.18 × 10^3^ (55.2 %)
**7**	49.32 × 10^3^ (0 %)	48.21 × 10^3^ (21.2 %)	37.65 × 10^3^ (23.7 %)	20.52 × 10^3^ (58.4 %)	19.20 × 10^3^ (61.1 %)	36.09 × 10^3^ (0 %)	31.89 × 10^3^ (11.6 %)	24.00 × 10^3^ (33.5 %)	21.78 × 10^3^ (39.7 %)	16.33 × 10^3^ (54.8 %)

## 3. Experimental Section

### 3.1. Materials and Methods

#### 3.1.1. Materials, Chemicals and Reagents

All the chemicals and solvents used in this study were obtained from Acros Organics, Thermo Fisher Scientific Company (New Jersey, NJ, USA), and of pure grades. Polyester fabrics were kindly supplied by Misr for Spinning and Weaving Company (Mahalla El-Kobra, Egypt). The dispersing agent Setamol WS was supplied by BASF (Nienburg, Germany). The bacterial strains from the American Type Culture Collection (ATCC) were purchased from MicroBioLogics Inc., (St. Cloud, MN, USA). The tested microorganism strains in ethanol were used as controls for biological experiments.

#### 3.1.2. Instrumentation

All melting points were measured by the capillary method on an electrothermal Gallenkamp melting point apparatus (Gallenkamp Co., London, UK) and were uncorrected. Elemental analyses were carried out at the Microanalytical Unit, National Research Centre, Giza, Egypt; the results were in satisfactory agreement with the calculated values. The IR spectra (KBr) were recorded in KBr disks by a Mattson 5000 FTIR spectrometer (Mattson Inst. Co., Madison, WI, USA) and not all frequencies are reported. The NMR spectra (^1^H and ^13^C) were acquired using a WP 300 spectrometer (Bruker Co., Billerica, MA, USA) at 300 MHz using TMS as an internal standard and DMSO-*d*_6_ as solvent. The colorimetric measurements for the dyed polyester fabrics were carried out using a reflectance spectrophotometer (GretagMacbeth CE 7000a, GretagMacbeth Co., Windsor, UK). Fastness to washing was carried out using the automatic Rotadyer launder (sponsored by the British Standard Institute—Society of Dyers and Colourists), fastness to perspiration was assessed according to the test sponsored by the (BSS), fastness to rubbing was carried out according to the standard method of testing (BSS) using a Crockmeter FD-17 apparatus (Electric Hungarian Co., Budapest, Hungary), fastness to sublimation was carried out using scorch tester M247 A (Atlas Electric Devices Co., Chicago, IL, USA) and fastness to light was carried out using the “Weather-o-meter” (Atlas Electric Devices Co.) according to the AATCC standard test method.

### 3.2. Synthesis

#### General Procedure for the Synthesis of 2-Amino-4-arylazo-thiazole Disperse Dyes **4**–**7**

A solution of aryl diazonium chloride was prepared by adding cold sodium nitrite solution (0.7 g in 10 mL H_2_O) to a cold suspension of aryl amine **2** and/or **3** (0.01 mol) in 3 mL concentrated HCl with stirring. The freshly prepared solution was added with continuous stirring to a cold solution (0–5 °C) of 2-aminothiazole **1** (1 g, 0.01 mol) in 30 mL ethanol and 4.0 g sodium acetate. The reaction mixture was stirred at 0–5 °C for 2 h, diluted with water, and the products collected by filtration. The obtained 2-amino-5-arylazothiazoles **4**–**7** were dried and recrystallized from ethanol.

*2-Amino-5-{[4-(carbamimidoyl-sulfamoyl)-phenyl]azo}thiazole* (**4**). Red solid, yield 74%, m.p. 220–221 °C. IR (KBr, cm^−1^): 3432, 3312, 3177 (NH_2_ and NH), 1640 (C=N). ^1^H-NMR (DMSO-*d*_6_, δ ppm): 7.05 (t, 1H, *J* = 7.15 Hz, NH), 7.59 (d, 2H, *J* = 7.80 Hz, Ar-H), 7.97 (d, 2H, *J* = 7.80 Hz, Ar-H), 8.36 (s, 1H, C-4 thiazole-H), 8.65 (d, 2H, *J* = 8.50 Hz, NH_2_), 9.40 (s, 2H, NH_2_), 11.35 (s, 1H, NH). ^13^C-NMR (DMSO-*d*_6_, δ ppm): 121.99 (2C), 129.51 (2C), 139.79, 144.88, 152.80, 155.20, 158.91, 173.40. Anal. Calcd. for C_10_H_11_N_7_O_2_S_2_ (325.37): C, 36.91; H, 3.41; N, 30.13,Found: C, 36.83; H, 3.45; N, 30.20.

*2-Amino-5-{[4-(carbamimidoyl-sulfamoyl)-phenyl] azo}-4-phenylthiazole* (**5**). Red solid, yield 77%, m.p. 241–241 °C. IR (KBr, cm^−1^): 3433, 3393, 3321 (NH_2_ and NH), 1639 (C=N). ^1^H-NMR (DMSO-*d*_6_, δ ppm): 6.94 (s, 1H, NH), 7.15–7.50 (m, 7H, Ar-H), 7.78 (d, 2H, *J* = 7.80 Hz, Ar-H), 8.48 (s, 2H, NH_2_), 9.18 (s, 2H, NH_2_), 11.37 (s, 1H, NH). ^13^C-NMR (DMSO-*d*_6_, δ ppm): 121.46 (2C), 126.55 (2C), 128.21, 129.38 (2C), 130.86 (2C), 137.32, 140.64, 144.32, 151.08, 154.56, 160.27, 174.38. Anal. Calcd. For C_16_H_15_N_7_O_2_S_2_ (401.47): C, 47.87; H, 3.77; N, 24.42, Found: C, 47.73; H, 3.84; N, 24.50.

*2-Amino-5-{[4-(pyrimidin-2-yl-sulfamoyl)-phenyl] azo}thiazole* (**6**). Red solid, yield 80%, m.p. 234–235 °C. IR (KBr, cm^−1^): 3351, 3254, 3146 (NH_2_ and NH), 1625 (C=N). ^1^H-NMR (DMSO-*d*_6_, δ ppm): 6.80 (s, 1H, NH), 7.17 (d, 1H, *J* = 7.50 Hz, C-5 pyrimidine-H), 7.32 (d, 2H, *J* = 7.80 Hz, Ar-H), 7.63 (d, 2H, *J* = 7.80 Hz, Ar-H), 7.96 (d, 2H, *J* = 8.80 Hz, C-4 and C-6 pyrimidine-H), 8.25 (s, 1H, C-4 thiazole-H), , 9.79 (s, 2H, NH_2_). ^13^C-NMR (DMSO-*d*_6_, δ ppm): 112.82, 122.48 (2C), 127.75 (2C), 131.84, 138.79, 140.11, 142.68, 156.21 (2C), 167.86, 173.36. Anal. Calcd. For C_13_H_11_N_7_O_2_S_2_ (361.40): C, 43.20; H, 3.07; N, 27.13, Found: C, 43.38; H, 3.15; N, 27.24.

*2-Amino-4-phenyl-5-{[4-(pyrimidin-2-yl-sulfamoyl)-phenyl]azo}thiazole* (**7**). Red solid, yield 72%, m.p. 181–182 °C. IR (KBr, cm^−1^): 3463, 3372, 3348, 3234 (NH_2_ and NH), 1625 (C=N). ^1^H-NMR (DMSO-*d*_6_, δ ppm): 6.84 (d, 1H, *J* = 7.50 Hz, C-5 pyrimidine-H), 7.11 (s, 1H, NH), 7.27–7.61 (m, 11H, Ar-H), 7.83 (d, 2H, *J* = 8.80 Hz, C-4 and C-6 pyrimidine-H), 8.62 (d, 2H, *J* = 8.50 Hz, NH_2_). ^13^C-NMR (DMSO-*d*_6_, δ ppm): 113.64, 123.71 (2C), 127.06 (2C), 128.46, 129.18 (2C), 130.69 (2C), 135.74, 136.62 138.08, 141.26, 144.59, 157.27 (2C), 167.38, 172.91.Anal. Calcd. For C_19_H_15_N_7_O_2_S_2_ (437.50): C, 52.16; H, 3.46; N, 22.41, Found: C, 52.02; H, 3.57; N, 22.57.

### 3.3. Disperse Dyeing Application

#### 3.3.1. Dyebath Preparation Dyeing Procedure

The synthesized disperse dyes **4**–**7** under investigation were applied to polyester fabrics (0.04 g dye/2 g fabric; o.w.f. 2%) by a convenient method for dyeing polyester fabrics in the laboratory at 130 °C and high-pressure (24–30 psi). A dispersion of dye was formed by dissolving the proper quantity of dye in 1 mL of acetone as solvent. 1% Setamol WS was added dropwise with continuous stirring to the dye bath as anionic dispersing agent. The liquor ratio was 20:1, and the pH was adjusted to 5.5 using aqueous acetic acid. Then, the wetted-out polyester fabrics were added to the dyebath. Dyeing process was accomplished by rising the dyebath temperature to 130 °C, at time intervals of 3 °C/min, the dyeing process was left for 60 min. After cooling to 50 °C, the dyed fabrics were rinsed with cold water and then reduction cleared using a mixture of 1 g/L sodium hydroxide and 1 g/L sodium hydrosulfite for 10 min. at 80 °C. The dyed fabrics were finally rinsed again with hot water followed by cold water and left for air drying.

#### 3.3.2. Dyeing Characteristics on Polyester Fabrics

##### Color Fastness Tests

The color fastness properties of the dyed fabrics to washing, perspiration, rubbing, sublimation and light were evaluated using standard methods [17]. The staining of adjacent cotton and nylon fabrics was assessed using the grey scale: 1—poor, 2—fair, 3—moderate, 4—good and 5—excellent, while the light fastness properties were scaled from 1–8 on the blue scale.

##### Color Properties of the Dyes on Polyester

A GretagMacbeth CE 7000a reflectance spectrophotometer (D65 illumination, 10° observer) was used for the colorimetric measurements on the dyed samples. K/S values given by the reflectance spectrometer were calculated at λ_max_ and were directly correlated with the dye concentration on the dye substrate according to the Kubelka–Munk equation: K/S = (1 − R)^2^/2R, where K = absorbance coefficient, S = scattering coefficient, R = reflectance ratio.

### 3.4. Biological Assessment

#### 3.4.1. Test-Pathogens

The antibacterial activity tests were performed against five bacteria strains: Gram-positive (*Staphylococcus aureus* ATCC 25923 and *Streptococcus pyogenes* ATCC 19615) and Gram-negative (*Salmonella typhi* ATCC 6539, *Pseudomonas aeruginosa* ATCC 27853 and *Klebsiella pneumoniae* ATCC 700603), as well as *Candida albicans* ATCC 90028 for the antifungal activity test. The microorganisms under test were grown onto Sabouraud agar. The microbial strains were conserved in Brain Heart Infusion medium (BHI) at −20 °C. Three hundred µL of each stock-culture was added to 3 mL of BHI broth. Cultures were kept for 24 h at 37 °C ± 1 °C and the purity of cultures was checked after 8 h of incubation. After 24 h of incubation, bacterial suspension was diluted with sterile sodium chloride solution, and bacterial suspension was diluted with BHI broth to a density of approximately 10^9^ colony forming unit/mL (CFU/mL) [21].

#### 3.4.2. Antimicrobial and Antifungal Activity

The antimicrobial activities of the dyes **4**–**7** were assessed *in vitro* using the agar diffusion method [22]. In this method, the medium was incubated with freshly prepared cells for each tested bacteria or fungi. After solidification of the agar, a number of clean disks were immersed into the solvents (negative controls) or extract solutions and located on the plates. After incubation at 37 °C for 24 h, the antimicrobial activity was assessed in term of diameter of the inhibition zone. Each test was repeated three times and the average results (mm of zone of inhibition) is tabulated.

#### 3.4.3. Minimum Inhibitory Concentration (MIC)

The selected bacteria for MIC were *Staphylococcus aureus* ATCC 25923 and *Salmonella typhi* ATCC 6539. MIC of four different samples was calculated by the two-fold serial dilution method [22,23]. The levels dose of 5, 10, 60, 150 µL/mL were utilized for MIC evaluation. 5 µL of standardized suspension of tested bacteria (10^8^ CFU/mL) well was used for 1 mL of the broth. The test tubes were incubated at 37 °C for 24 h. The growth was calculated by spectrophotometer at 600 nm. MIC for bacteria was determined as the lowest concentration of the compound inhibiting the visual growth of the test cultures on the agar plate. Each test in this experiment was repeated three times and the average results in term of (mm of zone of inhibition) was tabulated.

#### 3.4.4. Scanning Electron Microscopy

Scanning electron microscopy (SEM) was used to evaluate the susceptible bacteria that is subjected to the synthetic products. A Small piece of agar is cut from the inhibition zone and fixed in a glutaraldehyde solution 3% (*v*/*v*) buffered with 0.1 M sodium phosphate (pH 7.2) for 1 h at 25 °C. Later on, the samples are washed with a buffer of sodium phosphate. Then, all pieces were then post-fixed in 1 % (*w*/*v*) osmium tetroxide (OsO_4_) for 1 h and then washed again in buffer. Dehydration steps were performed using propylene oxide. The specimens were left for drying and were mounted onto stubs using double-sided carbon tape, and then were coated with a thin layer of gold by a Polaron SC 502 sputter coater. All of them were observed on a JSM 6060 LV Scanning Electron Microscope (Jeol Inc., Peabody, MA, USA) [24].

## 4. Conclusions

A series of novel 2-amino-5-arylazothiazole disperse dyes was synthesized. The newly synthesized dyes were applied as disperse dyes for dyeing polyester fabric, where they exhibited good dye ability and fastness properties. The novel compounds displayed broad-spectrum antimicrobial efficiency against selected pathogenic Gram-positive and Gram-negative bacteria, as well as fungi. The remarkable activity against Gram-negative bacteria of the newly synthesized dyes will be subjected to further study for the action mechanism of each compound.

The antimicrobial activity of the synthesized dyes does not affect the dyeing properties when applied on fabrics but it is highly interesting to dye fabrics with effective antibacterial agents. Processes utilizing textile engineering and chemical technologies to improve fabrics that function on human skin have evolved over the centuries. Similar approaches are needed in fiber science to improve the quality and performance of health care fabrics.

## Supplementary Materials

Supplementary materials can be accessed at: http://www.mdpi.com/1420-3049/21/1/122/s1.
