# Supplementary material for: Synthesis of Some Novel 2-Amino-5-arylazothiazole Disperse Dyes for Dyeing Polyester Fabrics and Their Antimicrobial Activity
Source: Molecules. 2016 Jan 21;21(1):122. doi: 10.3390/molecules21010122 (PMC6274149; doi:10.3390/molecules21010122)
Supplement: Supplementary file 1 [file molecules-21-00122-s001.pdf]

Supplementary Materials: Synthesis of Some Novel 2-Amino-5-arylazothiazole Disperse Dyes for Dyeing Polyester Fabrics and Their Antimicrobial Activity

Hatem E. Gaffer, Moustafa M. G. Fouda and Mohamed Khalifa

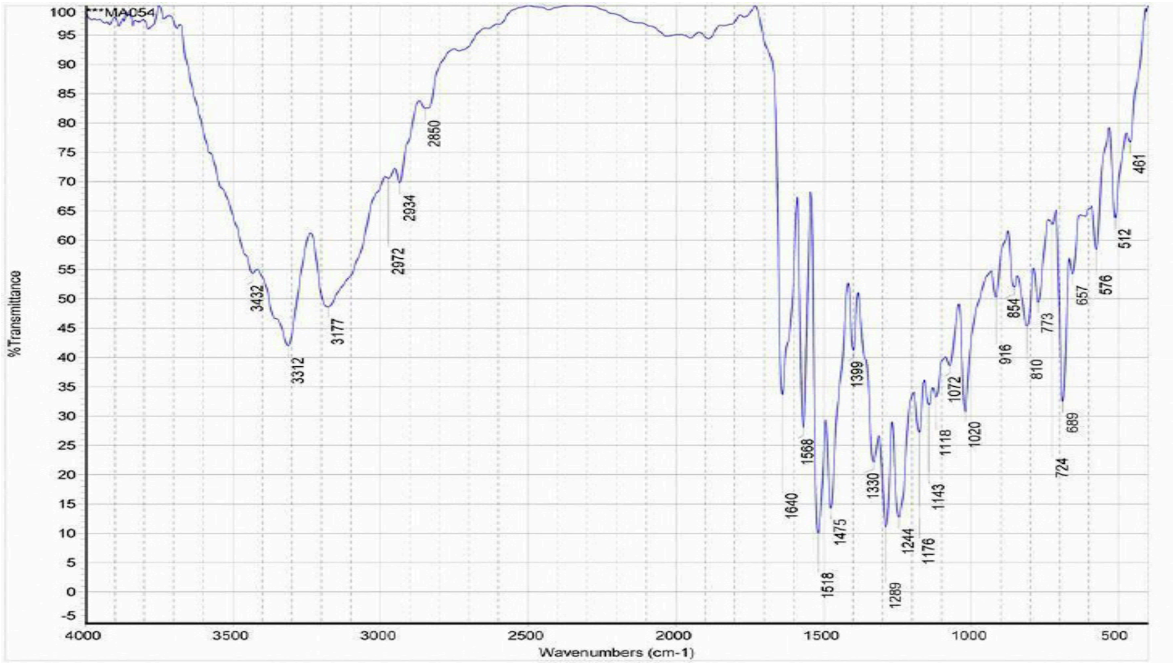

Figure S1. IR spectrum of compound (**4**).

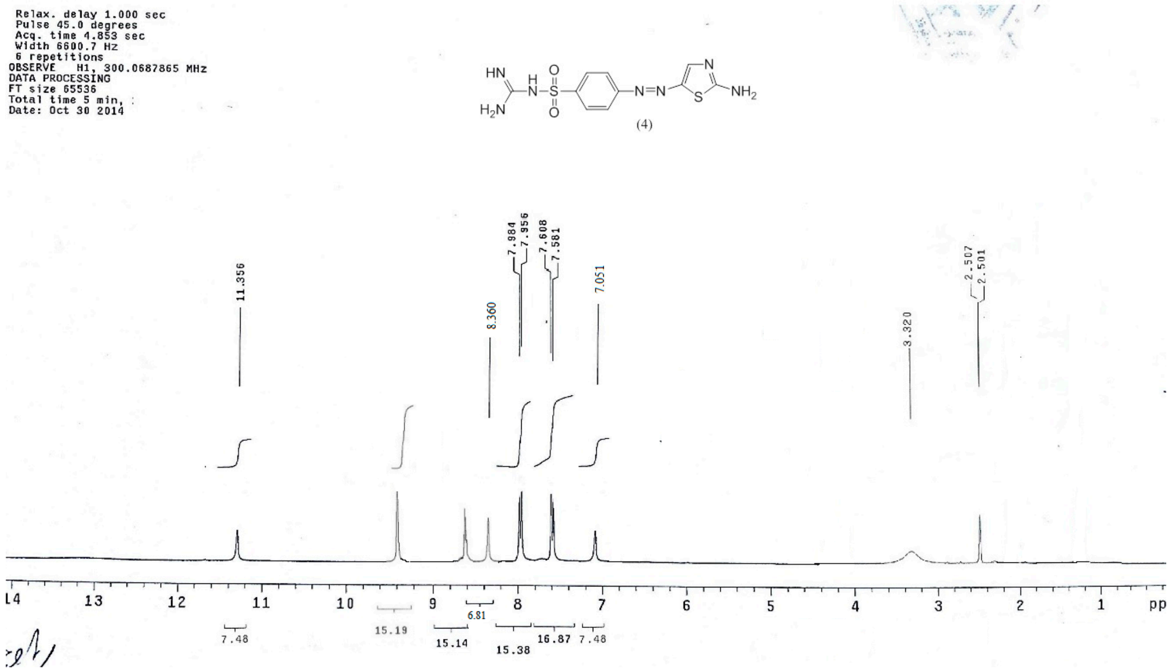

Figure S2. ^{1}H-NMR spectrum of compound (**4**).

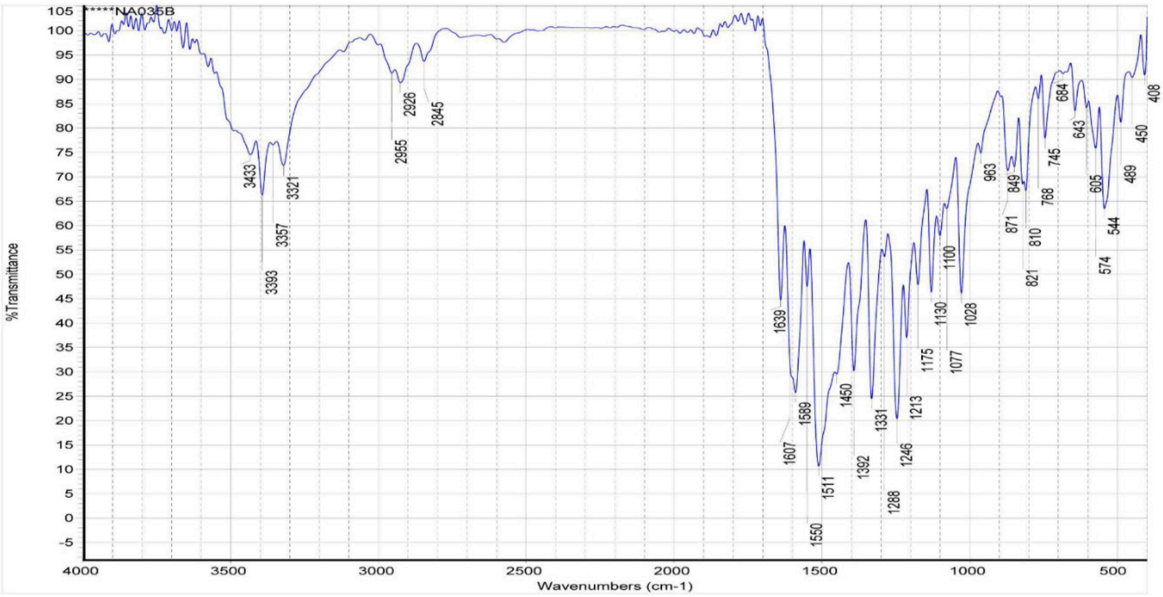

Figure S3. IR spectrum of compound (**5**).

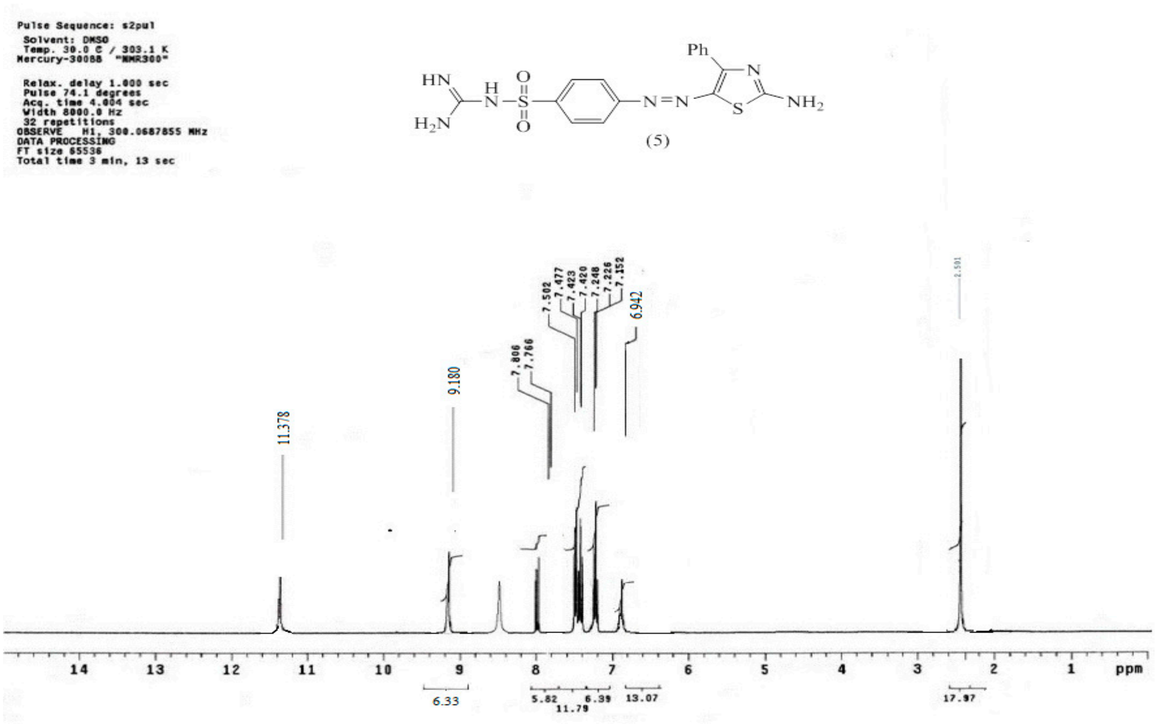

Figure S4. ^{1}H-NMR spectrum of compound (**5**).

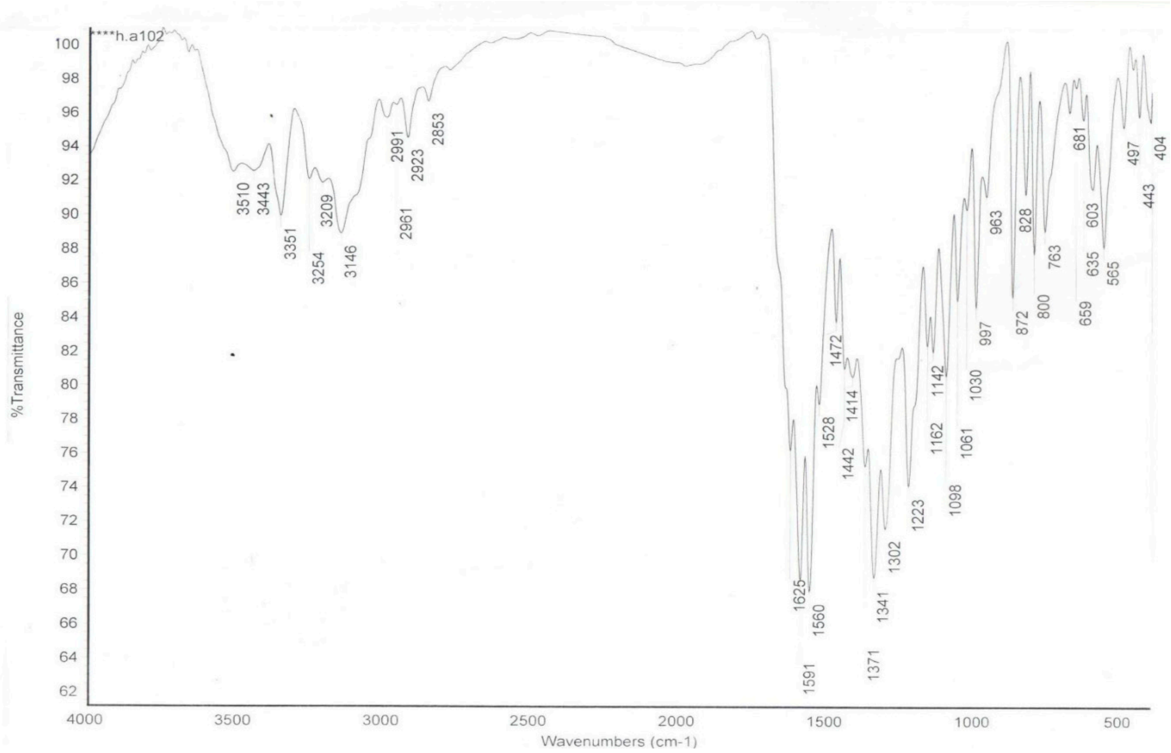

Figure S5. IR spectrum of compound (**6**).

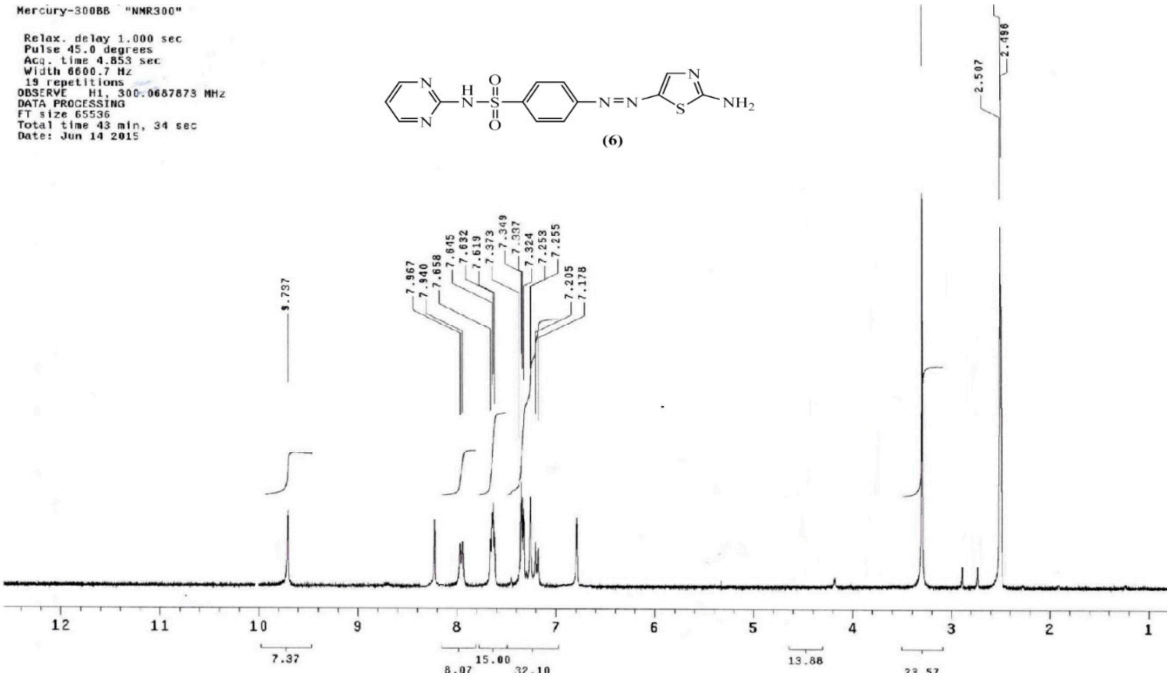

Figure S6. ^{1}H-NMR spectrum of compound (**6**).

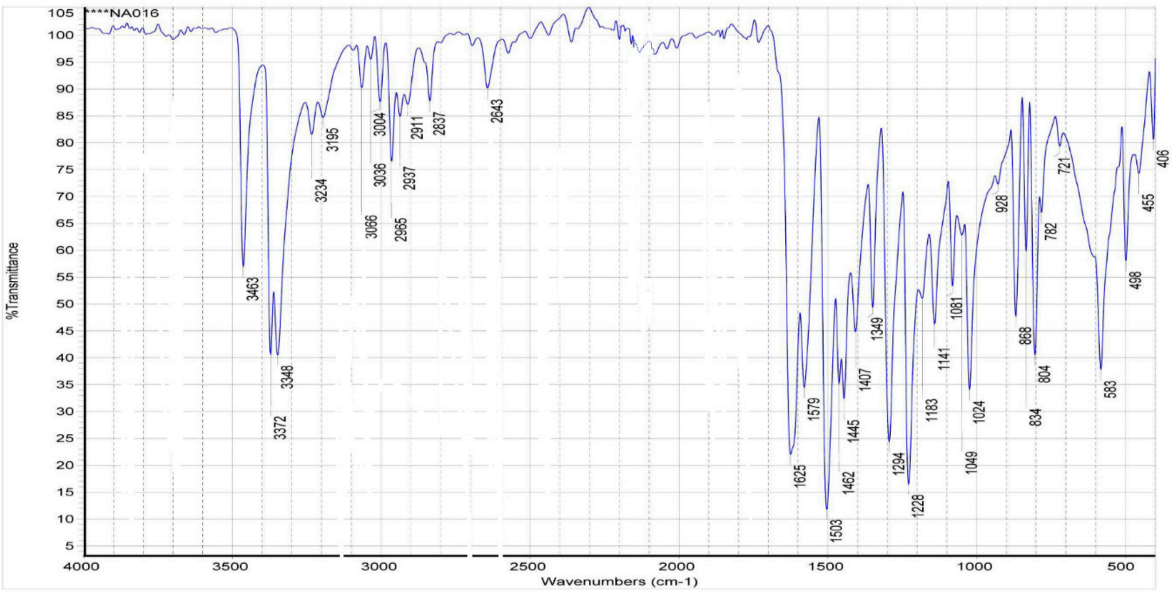

Figure S7. IR spectrum of compound (**7**).

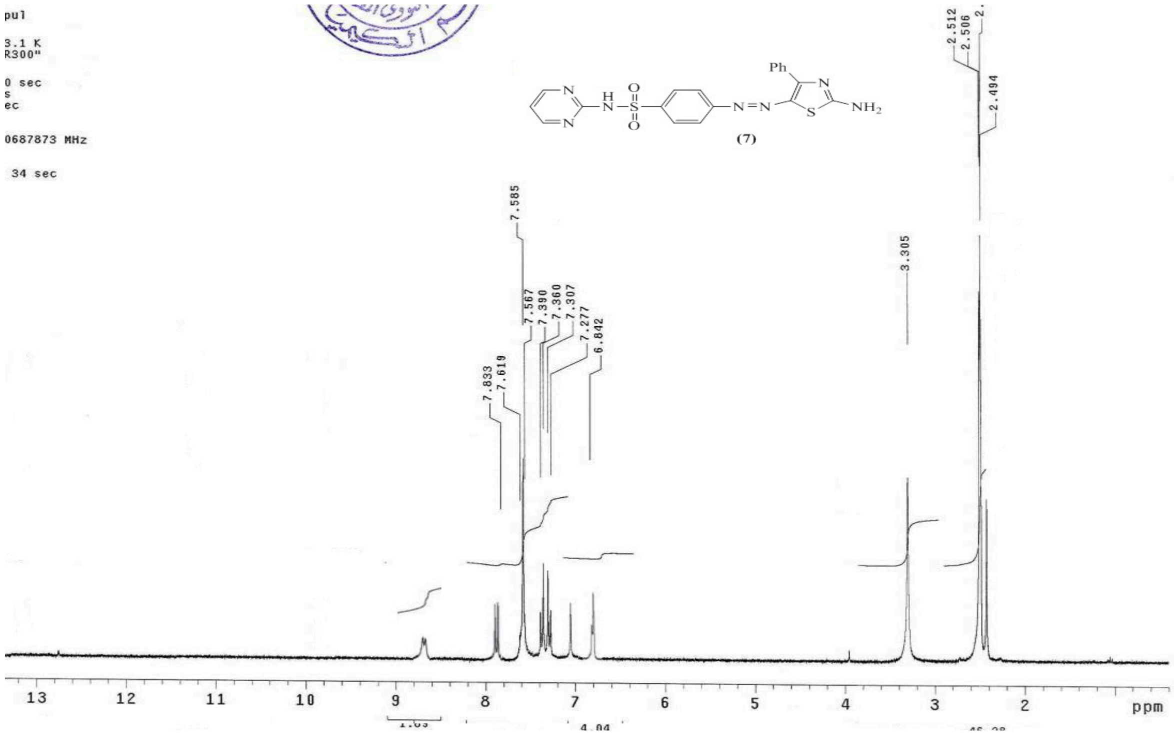

Figure S8. ^{1}H-NMR spectrum of compound (**7**).
